# Value systems of artificial intelligence and university students: theoretical dominance in large language models and religious priority in humans

**DOI:** 10.3389/fpsyg.2026.1755145

**Published:** 2026-04-21

**Authors:** Nabil Saleh Sufyan, Sami M. Alshehri, Ahmed Ali Teleb, Adel Abbady, Ahmed Ali Al Awad Asiri, Nilly H. Elamrousy, Mohamad A. S. Khasawneh

**Affiliations:** 1 Department of Psychology, College of Education, King Khalid University, Abha, Saudi Arabia; 2 Department of Teaching and Learning, College of Education, King Khalid University, Abha, Saudi Arabia; 3 Department of Special Education, College of Education, King Khalid University, Abha, Saudi Arabia

**Keywords:** artificial intelligence, large language models, Spranger’s classification, university students, value system

## Abstract

The rapid advancement of artificial intelligence (AI), particularly large language models (LLMs), raises critical questions about the value system these systems appear to reflect in comparison with human values. This study aimed to examine Spranger’s six value types (religious, social, theoretical, economic, political, and aesthetic) as manifested in three LLMs (OpenAI-o1, Gemini-2.0, and DeepSeek-V3), and to compare them with the value system of a sample of students at King Khalid University. A descriptive–comparative design was employed, administering the Study of Values to both groups: 214 students (male and female across academic levels) and the three LLMs, with repeated administrations to the latter to ensure test–retest reliability. Results indicated statistically significant differences in both the prominence and ranking of values across groups. Theoretical values consistently dominated in the LLMs, followed by social, aesthetic, and political values, with religious values ranking lowest. In contrast, students prioritized religious values, followed by theoretical values, while aesthetic values occupied the lowest ranks. Further, significant effects of gender and academic level were observed among students: religious values were more salient among females, theoretical values among males, and aesthetic values among undergraduates. These findings suggest that LLMs project value system shaped by their training data, rather than by human cultural or moral frameworks. The study highlights the importance of integrating culturally diverse value dimensions into AI development and calls for raising students’ awareness of using AI tools in ways aligned with human values. Effect-size estimates further indicated very large human–AI discrepancies, particularly in the religious (d = 2.21) and theoretical domains (d = 1.22).

## 1 Introduction

### 1.1 Background and significance

The rapid advancement of artificial intelligence (AI), particularly large language models (LLMs), has raised fundamental questions regarding the implicit value orientations embedded in AI-generated outputs.

In recent years, artificial intelligence (AI) has shifted from a theoretical concept to a transformative force reshaping psychology, education, and industry. Central to this transformation are large language models (LLMs), designed to simulate linguistic and cognitive processes in producing text and engaging in complex interactions (Haase and Hanel, 2023). Since its release in November 2022, OpenAI’s ChatGPT has demonstrated human-like linguistic performance, prompting rapid development of competing systems such as Google’s Gemini and China’s DeepSeek.

This growing reliance on AI raises ethical and societal concerns, particularly regarding the values embedded in its outputs (Kaya et al., 2024). In psychology, values are viewed as fundamental motives guiding behaviour and moral orientations (Zahran, 1984). Although LLMs lack self-awareness or subjective experience, their outputs may reflect implicit value patterns derived from training data and algorithms (Bodroža et al., 2023; Guo et al., 2023; Ye et al., 2024).

### 1.2 Research gap

A growing body of research has begun to benchmark large language models directly against humans using instruments originally developed to assess cognitive, social, and emotional functioning. Recent evidence suggests that models such as ChatGPT-4 can outperform psychology students and trainees on standardized measures of social intelligence and can exceed human norms on performance-based assessments of emotional awareness, achieving near-ceiling scores with strong expert-rated contextual appropriateness (Sufyan et al., 2024; Elyoseph et al., 2023). Parallel work in high-stakes academic and professional domains, including medical licensing examinations, university mathematics admissions tests, and histology assessments, has likewise shown that GPT-based systems may match or surpass average student performance when the item is treated as the unit of analysis (Meyer et al., 2024; Udias et al., 2024; Mavrych et al., 2025).

Despite these advances, the available literature has remained focused primarily on cognitive performance, emotional recognition, or accuracy in domain-specific testing. Considerably less attention has been devoted to the question of whether large language models exhibit stable value-related output patterns that can be meaningfully compared with human value priorities. This gap is consequential, because values are not peripheral psychological attributes; rather, they shape judgment, preference, decision-making, and social interpretation across educational, cultural, and interpersonal contexts. As AI systems are increasingly used in settings involving guidance, evaluation, and meaning-making, understanding the value-related tendencies reflected in their outputs becomes both theoretically important and practically necessary.

This gap is particularly evident in relation to classical psychological frameworks of human values. Although value theory has long occupied a central place in personality, social, and cultural psychology, relatively few studies have applied established value models to contemporary AI systems in a way that permits structured comparison with human respondents. Moreover, when such comparisons are attempted, they often risk anthropomorphic overinterpretation by implying that AI systems possess internal beliefs, intentions, or enduring value structures analogous to those of humans. A more conceptually disciplined approach is therefore needed—one that treats AI responses as patterned outputs generated under standardized prompting conditions, while still allowing psychologically meaningful comparison with human value priorities.

The present study addresses this gap by examining value systems in three prominent large language models—OpenAI-o1, Gemini-2.0, and DeepSeek-V3—and comparing their output patterns with the value priorities of students at King Khalid University. Specifically, the study draws on Spranger’s six value types—religious, theoretical, social, political, economic, and aesthetic—to explore areas of convergence and divergence between AI-generated and human value priorities. By doing so, the study aims to clarify the extent to which AI aligns with, or diverges from, human value orientations, and to contribute to ongoing discussions on culturally informed AI development, interpretive caution, and the responsible use of AI in psychological and social contexts.

Taken together, the foregoing review and identified research gap provide the foundation for the present investigation. Accordingly, the study was designed to examine value-related patterns in selected large language models and to compare them with the value priorities of university students within a structured psychological framework.

### 1.3 Objectives

This study aims to:

1. Compare value systems of three LLMs (ChatGPT-o1, Gemini-2.0, DeepSeek-V3).
2. Compare AI value profiles with human students.
3. Examine gender and academic level differences in human values.
4. Analyze convergence/divergence in value ranking patterns.

### 1.4 Research hypotheses

- There are statistically significant differences in value system among university students according to gender and academic level.
- There are statistically significant differences in value system among large language models (ChatGPT, Gemini, and DeepSeek) depending on model type.
- There are statistically significant differences in value system between students and large language models (ChatGPT, Gemini, and DeepSeek).
- Theoretical and social values are expected to rank highest in large language models, while religious and aesthetic values appear in the lowest ranks. In contrast, religious and social values are expected to rank highest among university students overall, with aesthetic values occupying the lowest ranks. Economic and political values are expected to fall in the middle range for both groups. Theoretical values are anticipated to vary among students, ranking differently between undergraduate and postgraduate levels.

### 1.5 Research delimitations

This study is delimited to the examination of values according to Spranger’s (1928) classification, conceptualized as interests, preferences, and judgements, and restricted to six value types: political, social, theoretical, religious, economic, and aesthetic. The scope is further limited to three large language models—ChatGPT-O1, Gemini-2.0, and DeepSeek-V3—tested during the period from 1 December 2024 to 30 January 2025. On the human side, the study is confined to male and female students enrolled in the College of Education at King Khalid University, across academic levels ranging from undergraduate to doctoral studies, during the 2024–2025 academic year.

Boundary conditions and transferability. Because the human sample is drawn from one university context (College of Education, KKU), the human value hierarchy should be interpreted as context-bounded and not assumed to represent all university students or cultures. In addition, administering the instrument in Arabic improves comparability with the human sample but may introduce cross-lingual effects for models whose training data are unevenly distributed across languages. Finally, while the reference base prioritizes peer-reviewed sources, a limited number of recent preprints are cited to reflect fast-evolving evidence in LLM research; these should be re-evaluated as peer-reviewed versions become available. Moreover, because commercial LLM providers routinely update or retire model versions, the exact model snapshots used during the data-collection window may no longer remain accessible, which can limit exact reruns on identical versions.

### 1.6 Challenges and mitigation strategies

One of the main challenges in this study concerned the stability of AI responses, which raised concerns of either inconsistency or exaggerated uniformity. This issue was addressed by administering the measures to the models repeatedly, at different times and across separate sessions using the same standardized procedure, to enhance test–retest reliability. Another challenge was the relatively small sample size of human participants. While this limitation may reduce the generalizability of the findings, it does not appear to undermine the accuracy or validity of the results obtained. We also recognise prompt sensitivity (i.e., shifts in model outputs due to small wording changes) as a methodological threat; this was mitigated by using a fixed prompt template, enforcing an A/B-only format, and repeating administrations across separate sessions.

## 2 Methods

### 2.1 Research design

The present study employed a descriptive–comparative design to examine the level and ranking of values among participants and to compare these across human and AI groups. This design was deemed appropriate for studies requiring the description of data, group comparisons, and analysis of potential differences between heterogeneous populations.

### 2.2 Population and participants

#### 2.2.1 The “human” participants are university students

Human participants. The human population consisted of students from the College of Education at King Khalid University, including both male and female students enrolled in closely related educational disciplines (psychology, curriculum and instruction, and educational administration). All academic levels were represented (undergraduate, master’s, and doctoral). A stratified random sampling method was used, yielding a total of 214 participants (134 males, 80 females). Given their shared educational focus, participants were considered comparable in disciplinary background. Table 1 presents the distribution of the human sample by gender and academic level.

**Table 1:** Distribution of the human sample by gender and academic level.

Gender/Academic level	Undergraduate	Master’s	Doctoral	Total
Male	56	29	49	134
Female	11	37	32	80
Total	67	66	81	214

#### 2.2.2 Artificial “participants” (LLMs)

The artificial “participants” in this study were three large language models (LLMs): ChatGPT-o1 (OpenAI), Gemini-2.0 (Google), and DeepSeek-V3 (DeepSeek). All models were accessed through their official web-based interfaces using the most recent publicly available versions during the data collection period (1 December 2024–30 January 2025).

To maximise comparability between human and AI data, the Study of Values (SOV) was administered to each LLM The Arabic version of the SOV—the same version administered to the students—was presented item by item to the models, with minimal adaptations to fit a text-only interaction and to enforce the forced-choice response format.

For each administration (“run”), a new conversation was initiated with the model to avoid contamination by prior context. At the beginning of each run, the model received a brief instruction describing the task and specifying that it must select only one of the response options per item (see Appendix 1 for the exact text of the prompts). Each SOV item was then presented with its two alternatives labelled (A) and (B), and the model was explicitly instructed to respond using a single letter (A or B) without explanation. When a model produced an output that did not conform to this response format (e.g., full sentences, multiple options), the item was immediately re-presented with a reminder of the response rule; if non-conformity persisted, the response was coded as missing for that item and excluded from scoring.

The models were administered the SOV multiple times in order to estimate the stability of their responses and to derive more robust model-level estimates. Specifically, the SOV was administered seven times to ChatGPT-o1, five times to Gemini-2.0, and five times to DeepSeek-V3. Each run was conducted in a separate session, at different times of day, to reduce the influence of transient system-level fluctuations and to approximate repeated measurements of a single system (i.e., system-level repeated measurements).

All prompts and SOV items were presented in Arabic to maintain linguistic consistency with the human sample and to approximate the same semantic content. No manual editing of model responses was performed apart from enforcing the A/B format. The resulting A/B responses were then scored using the same key and scoring procedures applied to the human participants, yielding six domain scores (theoretical, religious, social, political, economic, aesthetic) for each run of each model.

Prompt sensitivity and robustness. Because LLM outputs can shift with minor changes in instruction framing, we kept the prompt template and item wording strictly constant across runs and sessions. The inter-run correlations reported above (Supplementary Table S1) provide an initial stability check under this standardized protocol. Nevertheless, future replications should include prompt-perturbation analyses using minimally varied instruction templates and should report rank-order stability (e.g., Spearman correlations of value rankings) to quantify sensitivity explicitly.

### 2.3 Instruments

#### 2.3.1 Study of values (SOV)

The primary instrument employed in this study was the *Study of Values*, originally developed by Allport and Vernon (1931). The measure was translated into Arabic by Hanaa (1959) and subsequently adapted to the local cultural context by Sufyan (1995). The SOV has been widely used to assess value system and their hierarchical ranking, and its construct validity and reliability have been confirmed in multiple previous studies.

Content. The test consists of 45 forced-choice items, requiring respondents (human or AI) to prioritize one value while rejecting another simultaneously, thereby enhancing the precision of value differentiation (Hanaa, 1959). The SOV is grounded in Spranger’s classification, which classifies values into six domains:

- Social value: concern for others, viewing people as ends in themselves, characterized by empathy and compassion (Hanaa, 1959).
- Theoretical value: interest in knowledge and the discovery of truth, independent of practical or aesthetic considerations (Hanaa, 1959).
- Economic value: focus on utility, practicality, and evaluating objects and individuals according to their functional benefit (Hanaa, 1959).
- Aesthetic value: appreciation of beauty, harmony, and form, with evaluation of the world based on its structural composition (Hanaa, 1959).
- Political value: interest in power, leadership, control, influence, prestige, and engagement with public affairs.
- Religious value: Commitment to absolute spiritual or metaphysical standards and concern with transcendent or divine matters.

##### 2.3.1.1 Validity and reliability

Multiple lines of evidence support the validity of the SOV. In earlier local applications, the face validity of the Arabic version had already been examined among university students. For the current study, validity was reassessed with a pilot sample of 60 students (balanced by gender and academic level). Item-total correlations (Pearson) were computed, yielding coefficients ranging from 0.69 to 0.88 (Appendix 2), confirming significant item-domain consistency across all six value dimensions. All items were retained to ensure comprehensive coverage of value constructs.

Test–retest reliability was examined with the same subsample over a two-month interval. Coefficients were high across domains: theoretical (0.962), religious (0.945), political (0.903), economic (0.883), social (0.892), and aesthetic (0.860), indicating strong stability among human participants.

#### 2.3.2 AI-specific adaptation of the SOV

For administration to the LLMs, the Arabic SOV was converted into a structured text-based format compatible with conversational interfaces. Each forced-choice item was reformatted as a direct question followed by two clearly demarcated options (A and B). The substantive wording of the items and alternatives was preserved, and no changes were made to the content, polarity, or scoring key. The only adaptation consisted of adding explicit labels (e.g., “Option A,” “Option B”) and an instruction requesting the model to choose the option most consistent with the response pattern generated under the task instructions. This minimal adaptation was designed to preserve the psychometric properties of the SOV while making it executable in a human-AI interaction setting.

To verify the stability of AI responses, the instrument was administered repeatedly to each LLM. Within-model rank-order stability across the six value domains was strong for ChatGPT-o1 (Kendall’s W = 0.802, *p* < 0.001) and DeepSeek-V3 (W = 0.840, *p* < 0.001), and moderate for Gemini-2.0 (W = 0.448, *p* = 0.048). Dispersion indices (Mean, SD, CV, and 95% CIs) for each value domain are provided in Supplementary Tables S1, S2.

### 2.4 Data analysis

Data were analysed using IBM SPSS Statistics, Version 28. All tests were two-tailed with a nominal significance level of *α* = 0.05. In line with contemporary recommendations in psychological research, the emphasis was placed on effect sizes and confidence intervals, with *p*-values used as supplementary indicators rather than the sole basis for interpretation. Different analytic strategies were adopted for the human sample, the LLM outputs, and the human–AI comparisons to reflect the distinct nature of these data sources.

#### 2.4.1 Human sample

For the human participants, descriptive statistics (means, standard deviations, minimum and maximum scores, skewness, and kurtosis) were computed for each of the six value domains. The Shapiro–Wilk test was used to assess the normality of the value distributions in the student sample, supplemented by inspection of skewness, kurtosis, and histograms. Given the relatively large sample size, minor deviations from normality were treated as acceptable, and parametric procedures were retained with appropriate caution in interpretation.

To test Hypothesis 1 (gender and academic level effects on value orientations), separate two-way analyses of variance (ANOVAs) were conducted for each value domain, with gender (male, female) and academic level (undergraduate, master’s, doctoral) entered as between-subjects factors. For each ANOVA, we examined the main effects of gender and academic level as well as their interaction. Where omnibus tests were statistically significant, *post hoc* Tukey tests were performed to identify pairwise differences between academic levels. For all ANOVA results, we reported the F statistic, degrees of freedom, *p*-values, and partial eta squared (η^2^ₚ) as an index of effect size.

For bivariate comparisons involving only two groups (e.g., specific follow-up contrasts where appropriate), independent-samples t-tests were used. In such cases, Cohen’s d was calculated as a standardized measure of the magnitude of group differences and, when relevant, used as an effect-size metric in preference to relying solely on statistical significance.

#### 2.4.2 LLM outputs

Because repeated administrations to the same LLM (e.g., seven runs for ChatGPT, five for Gemini, and five for DeepSeek) represent repeated measurements on a single system rather than observations from independent individuals, the LLM data were treated primarily within a descriptive and reliability-oriented framework. For each model and each value domain, we computed: (a) the mean score across runs, (b) the standard deviation and range (minimum–maximum), (c) the coefficient of variation as an index of relative dispersion, and (d) 95% confidence intervals around the mean, using the t distribution appropriate for the number of runs per model.

To evaluate the stability of each model’s outputs across repeated administrations, we quantified rank-order agreement across runs using Kendall’s coefficient of concordance (W) over the six value domains, and we additionally report the mean and worst-case pairwise Spearman rank correlations (*ρ*) between runs as complementary stability indicators. Dispersion of run-level scores was summarized using the mean, SD, coefficient of variation (CV), and 95% confidence intervals (t-based) for each value domain. Full stability and dispersion results are reported in Supplementary Tables S1, S2.

To address Hypothesis 2 (differences across AI models), we conducted exploratory nonparametric comparisons using the Kruskal–Wallis test on the run-level scores for each value domain, treating runs as units of analysis within each model. Given that runs from the same model are not fully independent observations and that the number of runs per model is small, the Kruskal–Wallis results are reported and discussed as exploratory, sensitivity-type analyses. Substantive interpretation of differences among AI models relies primarily on the descriptive patterns and stability indicators (means, confidence intervals, and reliability estimates), rather than on formal hypothesis testing alone.

#### 2.4.3 Human–AI comparisons

For Hypothesis 3, which concerned differences between human and AI value profiles, the student sample was treated as a reference distribution against which the LLMs’ scores were compared. For each value domain and each LLM (and, where appropriate, for the pooled AI scores), we computed standardized mean differences by expressing the AI mean in relation to the student mean and standard deviation. Specifically, Cohen’s d was calculated using the student standard deviation as the denominator, thereby quantifying how many standard deviations the AI model’s mean lay above or below the human mean for each value domain. These effect sizes were interpreted as the primary indicators of the substantive magnitude of human–AI discrepancies.

In addition, independent-samples t-tests were conducted, comparing the human participants (*N* = 214) with the set of AI runs (e.g., the 17 scored LLM administrations combined) for each value domain. In these tests, the group variable contrasted students versus AI responses, and separate t-tests were run at the value-domain level. However, because the AI “cases” are repeated outputs from a small number of models rather than independent individuals, these inferential tests were treated as secondary, exploratory analyses aimed at assessing the robustness of the observed differences. Interpretation of human–AI contrasts therefore focused primarily on the direction and magnitude of Cohen’s d and the associated confidence intervals, with *p*-values from t-tests serving only as supplementary evidence.

Finally, to address Hypothesis 4 concerning value rankings, we examined the hierarchical ordering of the six value domains for students and for each AI model based on their mean scores. Rankings were summarized in tables and visualized in figures. We also computed descriptive indices of convergence and divergence in rank order (e.g., overlaps in the top-ranked and bottom-ranked values) between humans and AI models. These analyses were intentionally nonparametric and descriptive, reflecting the ordinal nature of rank data and our interest in global pattern similarity rather than fine-grained statistical testing of rank differences.

## 3 Results

### 3.1 Normality testing

The Shapiro–Wilk test was applied to examine the distribution of the six value domains among student participants. Descriptive statistics and normality results are presented in Table 2 Most values met the assumption of normality (*p* > 0.05), with the exception of the aesthetic value (*p* = 0.004). Given the adequate sample size, parametric tests were retained, with caution applied in interpreting the aesthetic value results.

**Table 2:** Descriptive statistics and Shapiro–Wilk test for checking the assumption of normal distribution of values.

Values	Mean	SD	Skewness	Kurtosis	Shapiro–Wilk	*p-*value
Theoretical	42.90	7.45	−0.101	−0.001	0.987	0.06
Religious	43.607	8.859	−0.289	−0.342	0.987	0.06
Social	37.748	6.184	0.055	−0.398	0.989	0.10
Aesthetic	33.393	7.881	0.455	−0.037	0.980	0.004
Political	41.486	6.599	−0.147	−0.415	0.991	0.20
Economic	40.860	7.125	−0.081	−0.075	0.992	0.31

### 3.2 Within-model stability across repeated LLM runs

Across repeated runs under the standardized protocol, ChatGPT-o1 exhibited strong agreement in the rank ordering of the six value domains (Kendall’s W = 0.802, χ^2^(5) = 28.083, *p* < 0.001), with a mean pairwise Spearman *ρ* of 0.769 (worst-case ρ = 0.462). DeepSeek-V3 similarly showed strong rank-order stability (W = 0.840, χ^2^(5) = 21.012, *p* < 0.001; mean ρ = 0.800; worst-case ρ = 0.585). Gemini-2.0 showed moderate stability (W = 0.448, χ^2^(5) = 11.192, *p* = 0.048), with lower pairwise agreement across runs (mean ρ = 0.311; worst-case ρ = −0.058), indicating greater sensitivity to transient fluctuations even under fixed instructions. Supplementary Table S2 summarizes dispersion (Mean, SD, CV, and 95% CIs) for each value domain within each model.

### 3.3 Hypothesis 1: gender and academic level effects

*There are statistically significant differences in value system among students according to gender and academic level.*

Two-way ANOVA was conducted for each value domain. *Post hoc* Tukey comparisons were performed where appropriate. Key results were as follows:

- Theoretical value: significant differences by academic level (*F* = 7.067, *p* = 0.001), with doctoral students scoring higher than undergraduates (Mean Difference = 4.33, *p* = 0.001). Significant gender differences also emerged (*F* = 11.534, *p* = 0.001), favouring males (Appendix 3).
- Religious value: Significant differences were observed by gender (*F* = 4.721, *p* = 0.031), with females scoring higher. No significant differences were found by academic level.
- Social, political, and economic values: No significant differences were observed by gender or academic level (*p* > 0.05).
- Aesthetic value: Significant differences by academic level (*F* = 4.723, *p* = 0.010), with undergraduates scoring higher than doctoral students (Mean Difference = 4.24, *p* = 0.003).

#### 3.3.1 Effect sizes

Effect sizes were computed for all statistically significant tests. The effect of academic level on theoretical values was of medium magnitude (η^2^ₚ = 0.063), and the effect of gender on theoretical values was also medium (η^2^ₚ = 0.052). For religious values, the effect of gender was small to medium (η^2^ₚ = 0.022). The effect of academic level on aesthetic values was of small-to-medium magnitude (η^2^ₚ = 0.043).

According to the Tukey HSD post hoc test, the significant difference was found only between bachelor’s and Ph.D. students, in favor of Ph.D. students, on the theoretical value. Appendix 3 presents the detailed results.

### 3.4 Hypothesis 2: differences across AI models

*There are statistically significant differences in value system among AI models (ChatGPT, Gemini, DeepSeek).*

The Kruskal–Wallis test revealed significant differences among models in the theoretical (H = 10.145, *p* = 0.006), social (H = 10.053, *p* = 0.007), and aesthetic values (H = 5.990, *p* = 0.050), favouring ChatGPT over the other models. No significant differences were observed in the economic (H = 2.006, *p* = 0.367), political (H = 5.089, *p* = 0.079), or religious values (H = 1.416, *p* = 0.493) (Appendix 4).

However, these were used only as exploratory diagnostics, given that they do not address direct differences between the LLMs themselves However, these analyses were treated as exploratory because the number of repeated runs within each model was limited and the run-level observations were not fully independent. Accordingly, they were not treated as the primary basis for evaluating Hypothesis 2.

Hypothesis 2. Instead, our main inferential focus for comparing the three AI models relied on human-referenced effect sizes, namely the standardized mean differences between GPT, Gemini, and DeepSeek expressed in units of the student standard deviation for each value domain (see Table 3). These effect sizes provide a more interpretable and psychometrically coherent index of between-model differences: GPT shows very large advantages over both Gemini and DeepSeek in the theoretical and social domains (exceeding 2–3 human SD units), medium-to-large advantages in the economic, political, and aesthetic domains, and a clear superiority over DeepSeek—but only a small edge over Gemini—in the religious domain. Because these human-scaled effects directly quantify the magnitude and practical significance of the discrepancies among models, our substantive conclusions regarding differences between LLMs are based primarily on the pattern and size of these standardized effects, with the Kruskal–Wallis test serving only as supporting, exploratory evidence.

**Table 3:** Human-referenced standardized differences between AI models across value domains.

Value domain	GPT mean	Gemini mean	DeepSeek mean	d (GPT–Gemini)	d (GPT–DeepSeek)	d (Gemini–DeepSeek)
Theoretical	57.00	40.20	37.00	2.26	2.68	0.43
Social	47.00	29.00	26.00	2.91	3.40	0.49
Economic	29.57	25.20	20.80	0.61	1.23	0.62
Political	35.14	28.80	27.40	0.96	1.17	0.21
Aesthetic	33.86	27.20	27.80	0.84	0.77	−0.08
Religious	26.00	23.40	11.80	0.29	1.60	1.31

Standardized differences (d) are expressed in units of the student sample’s standard deviation for each value domain.

### 3.5 Hypothesis 3: differences between humans and AI models

*There are statistically significant differences in value system between students and AI models (ChatGPT, Gemini, DeepSeek).*

Independent-samples (t) tests indicated significant differences between human and AI mean scores across most values (Appendix 5). The most salient results were:

- Religious value: students scored substantially higher than all three AI models.
- Theoretical value: AI models scored relatively higher than students, with differences varying by model.
- Social value: students demonstrated higher scores than certain AI models, with differences depending on the specific comparison.

#### 3.5.1 Effect sizes

For human–AI comparisons, standardized mean differences revealed very large effects in the religious domain (d = 2.21), large effects in the theoretical domain (d = 1.22), and medium effects in social values (d ≈ 0.55). Effect sizes in economic and political domains were small to medium (d = 0.25–0.35), while the aesthetic domain showed a large effect (d ≈ 0.85). These effect size estimates provide a more informative interpretation of group differences than reliance on *p*-values alone.

### 3.6 Hypothesis 4: ranking patterns

Theoretical and social values are expected to rank highest among AI models, while religious and aesthetic values rank lowest. In contrast, religious and social values are expected to rank highest among university students, with aesthetic values occupying the lowest ranks. Economic and political values are expected to remain in the middle range for both groups, while theoretical values vary among students according to academic level.

**Figure 1:**
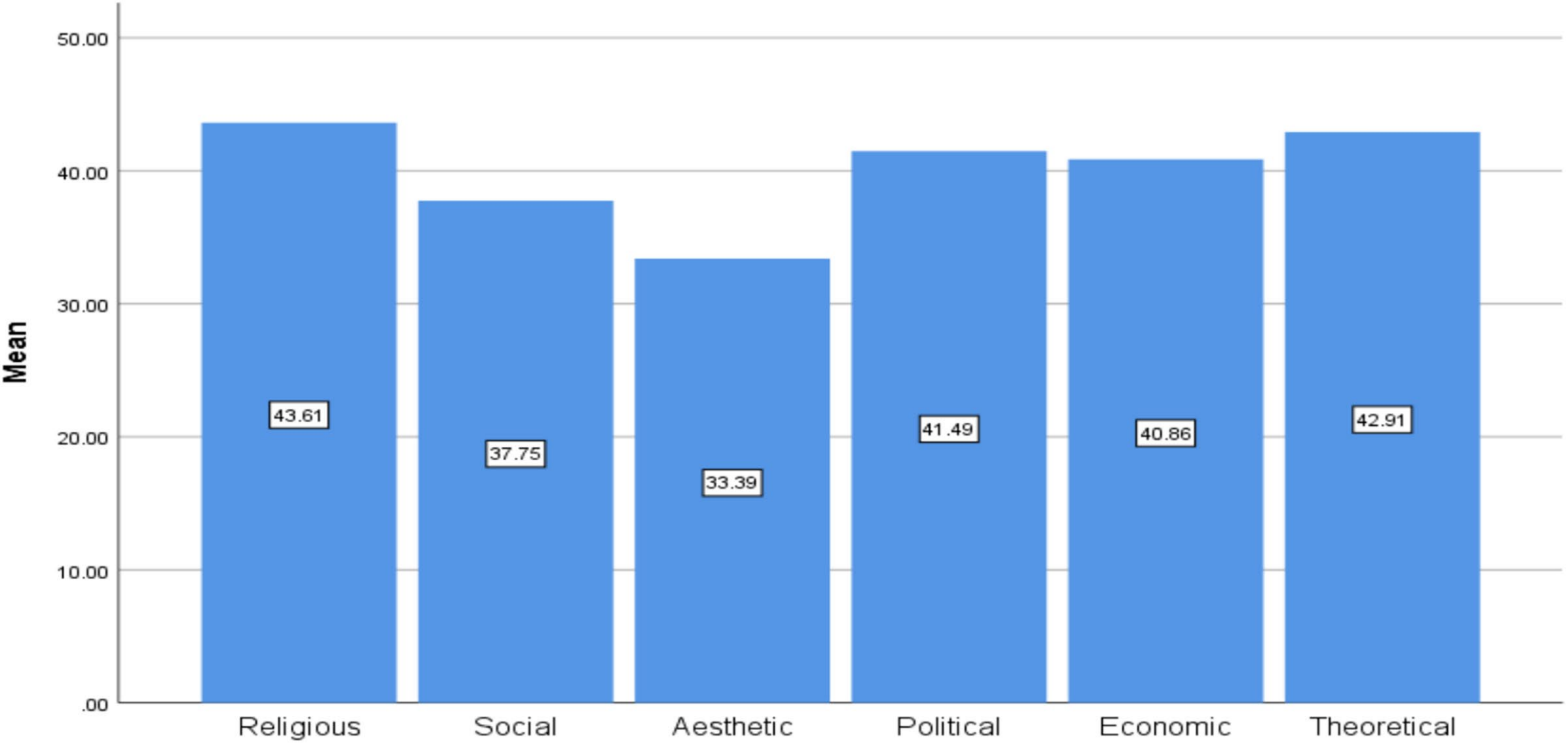
Ranking of the six value domains among university students overall.

**Figure 2:**
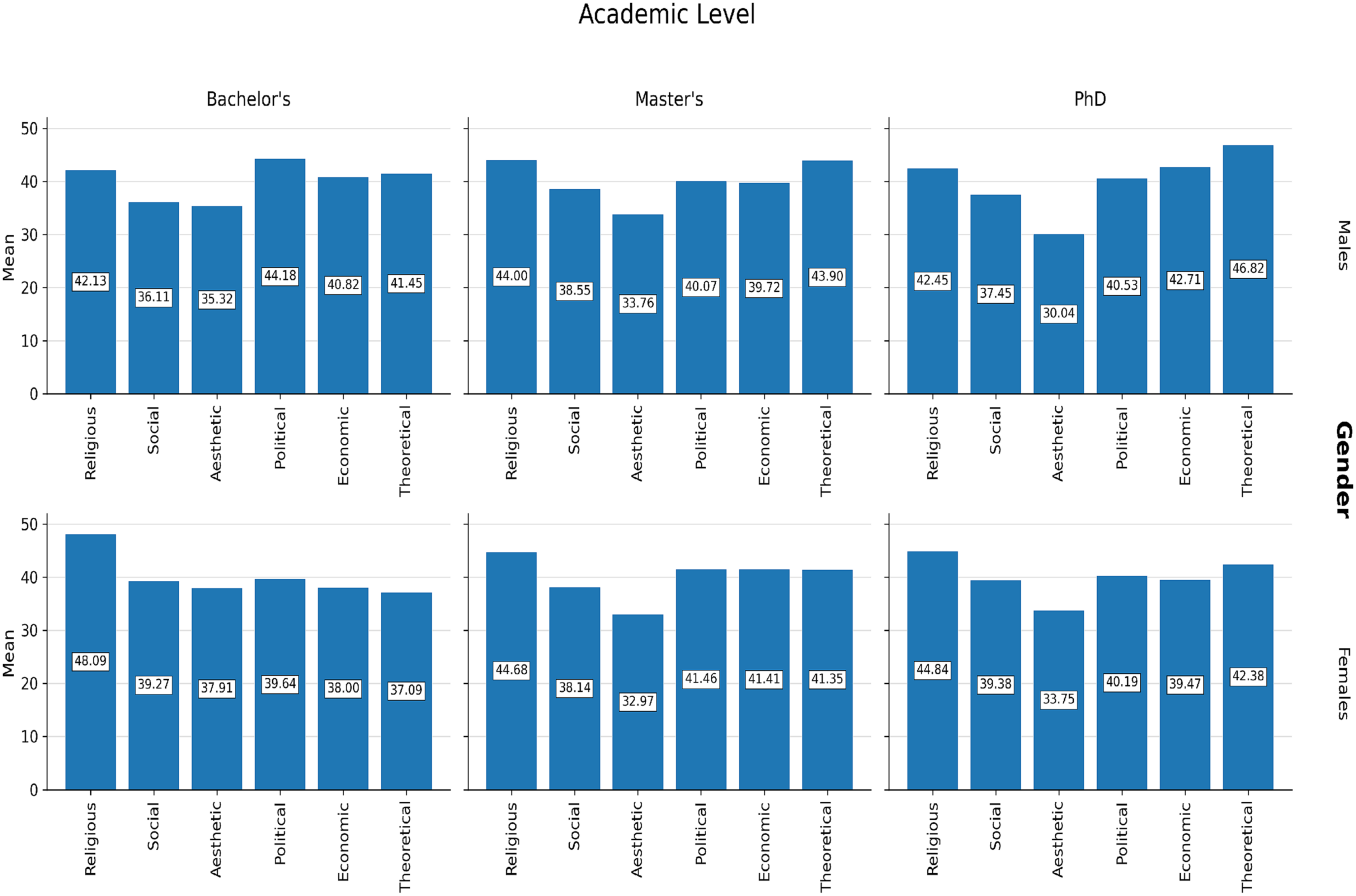
Ranking of the six value domains among university students by gender and academic level.

**Figure 3:**
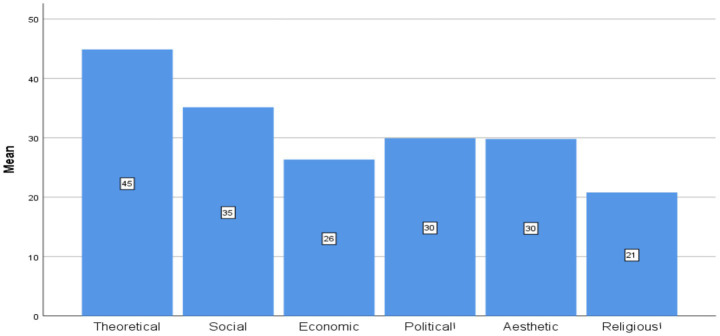
Ranking of the six value domains across large language models (LLMs) overall.

**Figure 4:**
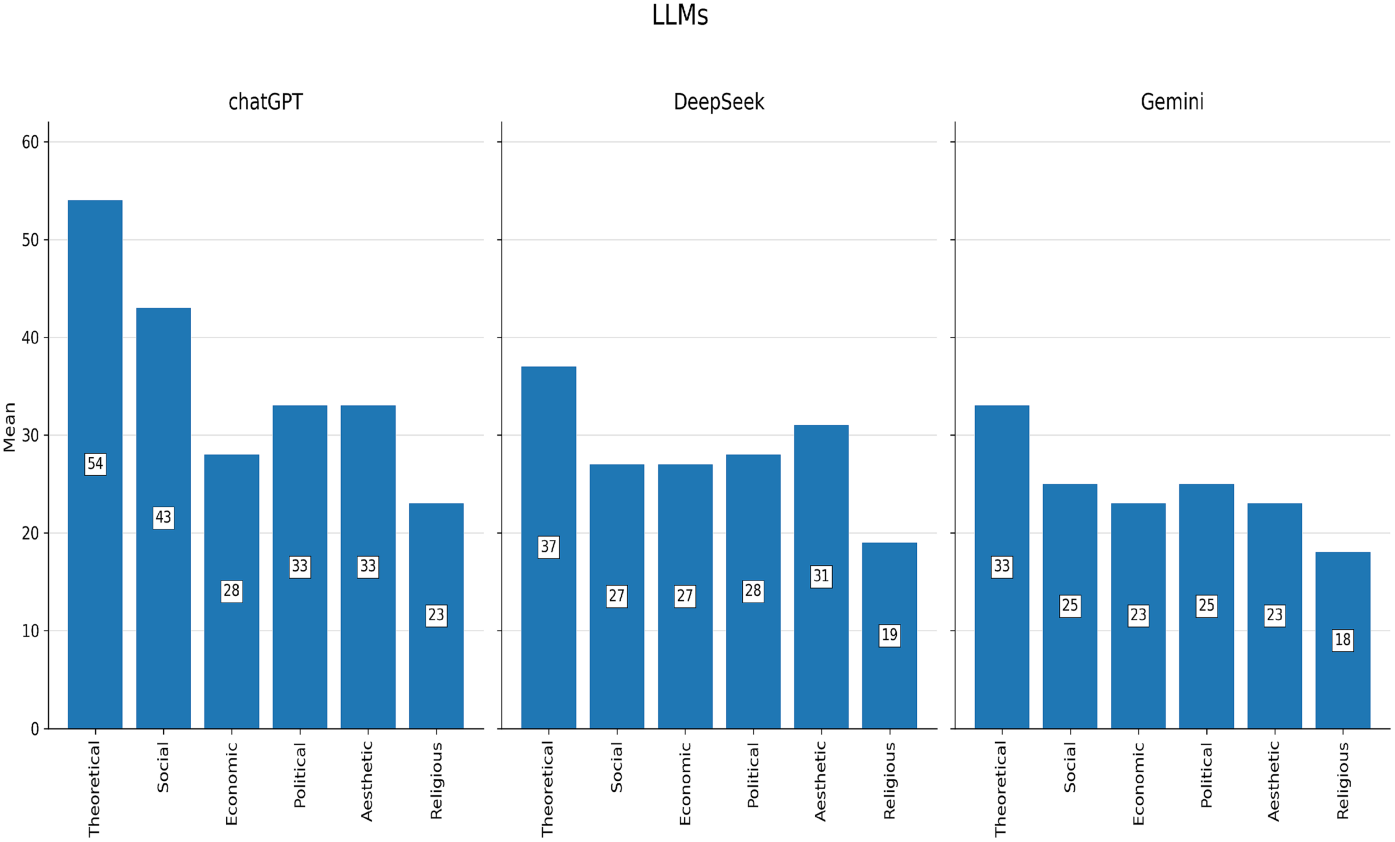
Ranking of the six value domains for each large language model (LLM) separately.

Ranking analyses were conducted based on mean scores for each value domain (Appendix 6; Figures 1–4).

AI models. Across all three LLMs, the theoretical value consistently occupied the highest rank, followed by social, aesthetic, and political values (with minor variation among models). Religious values consistently ranked lowest.

University students. Religious values ranked highest for the majority of students, with the exception of some doctoral participants who prioritized theoretical values. Aesthetic values ranked lowest overall, while political and economic values occupied middle positions.

Summary of rankings (Appendix 6):

- AI models (e.g., ChatGPT): 1. Theoretical, 2. Social, 3. Aesthetic, 4. Political, 5. Economic, 6. Religious.
- Students: 1. Religious, 2. Theoretical, 3. Political, 4. Economic, 5. Social, 6. Aesthetic.

Figures 1–4 illustrate these ranking patterns for students and AI models overall, as well as disaggregated by gender, academic level, and model type.

Overall, the distribution of values appears ordered as follows.

Religious values occupied the highest position, followed by theoretical, political, economic, social, and finally aesthetic values, which ranked lowest.

Across groups, female students tended to priorities religious values, whereas male students scored higher on theoretical values. Differences across academic levels also emerged, with doctoral students placing greater emphasis on theoretical values, whereas undergraduates showed relatively greater emphasis on religious and aesthetic values.

Figure 3 illustrates the distribution of the values. Theoretical values consistently occupied the highest rank, followed by social, aesthetic, and political values, with minor variation across models. Economic values appeared in the mid-range, whereas religious values were consistently ranked lowest.

Figure 4 clearly indicates that ChatGPT placed theoretical and social values at the top, with religious values at the bottom. Gemini showed a similar pattern but with slightly higher emphasis on political values. DeepSeek prioritised theoretical values most strongly, followed by aesthetic and social values, while again ranking religious values last. These variations highlight both the consistency of certain patterns (e.g., dominance of theoretical values) and model-specific differences in secondary value preferences.

Taken together, these findings provide the empirical basis for a broader interpretation of how value-related patterns differ between human participants and large language models. The following discussion situates these results within the literature on cultural values, AI alignment, and context-sensitive psychological interpretation.

## 4 Discussion

The findings reveal a clear value gap between large language models (LLMs) and university students, a gap that warrants close attention given the rapid advancement of AI and its growing influence across multiple domains of life. By systematically analysing the outputs of leading LLMs (ChatGPT, Gemini, DeepSeek) and comparing them with students’ value system at different academic levels, this study provides novel insights into how “values” may manifest in AI systems and raises critical questions about their alignment with human values. The results also underscore the importance of demographic, cultural, and social factors in shaping value system among individuals and point to the need for embedding cultural filters into AI systems to enhance their compatibility with prevailing societal values.

### 4.1 Human value differences

The results indicated significant differences in theoretical and aesthetic values across academic levels: doctoral students scored higher on theoretical values, whereas undergraduates scored higher on aesthetic values. This finding suggests developmental shifts in students’ value system as they progress through higher education, with greater emphasis on scientific inquiry and critical thinking at advanced stages. These results are consistent with previous research indicating that value rankings may change during university years (Bakr, 1975; Cox, 1989; Dobashi, 1976).

Gender differences also emerged, with males scoring higher on theoretical values and females scoring higher on religious values. These findings align with earlier studies highlighting the role of demographic and sociocultural factors—such as gender and educational level—in shaping value system (Al-Batsh and Abd al-Rahman, 1990; Allen, 1981; Abu al-Nil, 1985; Zahran and Serry, 1985; Abd al-Fattah, 1992; McGuiness-Biewitt, 1985). Specifically, prior research (Sufyan, 2002; Al-Suwwad and Al-Azirjawi, 1987; Hanaa, 1959) consistently reported higher theoretical values among males, while political values tended not to show significant gender-related differences (Al-Batsh and Abd al-Rahman, 1990; Allen, 1981). Similarly, economic values were previously found to favor males (Al-Suwwad and Al-Azirjawi, 1987).

In contrast, the present study found no significant gender differences in social values, with male and female students scoring similarly. This result diverges from much of the earlier literature, which typically found higher social values among females (Zahran and Serry, 1985). However, Abu Al-Nil (1985) reported contrary findings, suggesting that local cultural factors may shape gender patterns in social values. In the Saudi context, recent societal transformations may have contributed to the convergence of male and female scores on this dimension.

With regard to aesthetic values, the current findings showed higher scores among undergraduates compared with doctoral students. This may reflect age-related interests, as younger students tend to show greater concern with beauty and artistic appreciation, whereas doctoral students are more heavily engaged in research and professional pursuits. Previous research often reported higher aesthetic values among females (Sufyan, 2002; Al-Suwwad and Al-Azirjawi, 1987; Hanaa, 1959; McGuiness-Biewitt, 1985), though some studies (Allen, 1981) failed to detect gender effects.

Finally, religious values showed gender-based differences in favor of females in the present study. This result is consistent with some previous studies (Hunt, 1980; Dobashi, 1976), though others reported the opposite pattern, with higher religious values among males (Abd al-Fattah, 1992; Al-Suwwad and Al-Azirjawi, 1987; Sabḥān, 1975). Such inconsistencies across studies likely reflect differences in religious traditions, societal contexts, and historical periods.

### 4.2 Differences across LLMs

The results revealed statistically significant differences among LLMs in theoretical, social, and aesthetic values, favouring ChatGPT over Gemini and DeepSeek. This finding suggests that AI “values” are essentially reflections of the training data and the algorithms employed, and that variation in both the nature of the data and model architectures contributes to observable differences in outputs. As Bender et al. (2021) emphasised, LLMs operate on vast corpora of text using machine learning algorithms, without possessing self-awareness or lived experience. Although some models are developed in Western contexts (e.g., ChatGPT, Gemini) and others in Eastern contexts (e.g., DeepSeek), these results do not necessarily reflect cultural differences in training data, given the opacity of data sources. Rather, they may point to differences in design, optimisation, and filtering processes. The relatively stronger alignment of ChatGPT with human-like value structures may suggest a comparative advantage in “value balance,” though this remains an artefact of training rather than evidence of genuine value endorsement.

### 4.3 Human–AI divergence

Comparisons between human and AI groups demonstrated substantive differences in most value domains. The most striking was the religious value, which was consistently higher among students than all three LLMs. By contrast, theoretical values appeared higher in AI outputs compared with student means, albeit with some variation across models. Social values also showed differences, with students scoring higher in certain subgroups than some AI models. These findings highlight a fundamental divergence between human and AI value system, reflecting differences in the sources of formation—cultural socialization and lived experience for humans, versus statistical learning from large-scale text corpora for AI. Such divergences underscore the importance of critically examining the implicit priorities embedded in LLM outputs, particularly when these systems are used in sensitive psychological, educational, or social contexts (Haase and Hanel, 2023; Flint et al., 2022; Bodroža et al., 2023).

Recent literature further supports interpreting human–AI differences through agency, evaluation, and contextual responsiveness rather than task accuracy alone. Generative AI can expand exploratory thinking and cognitive diversity, yet its outputs remain conditioned by human design choices, governance structures, and deployment contexts (Krakowski, 2025). In higher education, AI may approximate human evaluative judgments, but it often moderates extreme scores and remains sensitive to task framing and response structure (Flodén, 2025). At the user level, AI decisions are frequently perceived as less fair and less comprehensible than human decisions unless explanatory mechanisms are provided (Shulner-Tal et al., 2025). Within education and science, these patterns highlight the importance of culturally responsive, ethically guided, and context-aware AI implementation (Dzogovic et al., 2024).

### 4.4 Value ranking analysis

The results showed that AI models consistently prioritized theoretical values, followed by social, aesthetic, or political values, while religious values ranked lowest. In contrast, religious values occupied the top rank among most students, with aesthetic values consistently ranking last and political and economic values positioned in the middle. This divergence reinforces the notion that values are not merely abstract beliefs but integral to personal identity and culture (Zahran and Serry, 1985). By contrast, AI models appear to reflect orientations closer to those dominant in scientific and technological domains, particularly within Western contexts.

### 4.5 The problem of “values” in AI

A central issue raised by this study is whether the term “values” can legitimately be applied to AI outputs. LLMs lack self-awareness and lived experience, yet their textual outputs reveal structured patterns in how they rank and prioritize concepts aligned with Spranger’s (1928) six value domains. Following Floridi and Chiriatti (2020), the behavioral outputs of AI systems provide a meaningful basis for value analysis, even if such rankings are not consciously held. An ongoing debate remains as to whether AI should embody human values or maintain neutrality, raising profound questions about AI ethics and developer responsibility (Taddeo and Floridi, 2018).

In line with critiques that describe LLMs as “stochastic parrots” that reproduce statistical patterns from their training data (Bender et al., 2021), we interpret the observed value rankings as output-level regularities shaped by pretraining corpora and post-training alignment, not as internally held convictions. Accordingly, our claims are confined to what the models express under a controlled psychometric prompt, and we avoid attributing intentionality, lived experience, or moral agency to the systems.

### 4.6 Cultural variables

The findings also highlight the significance of demographic, cultural, and social variables in shaping human value hierarchies. Results are consistent with prior research documenting the influence of gender and educational level on value system (Hanaa, 1959; Al-‘Umari and Nashwan, 1985; Bakr, 1975; Cox, 1989; Dobashi, 1976; Sufyan, 2002). The academic climate itself appears to reinforce certain value domains, as suggested by earlier studies (Al-‘Umari and Nashwan, 1985; Cantrell, 1976; McGuiness-Biewitt, 1985; Malkosh, 1996).

### 4.7 Need for cultural filters

This study calls for embedding “cultural filters” into AI systems to ensure greater alignment with the values of diverse user populations, especially in societies with strong religious and cultural identities (Li et al., 2022). Such an approach reflects growing recognition of cultural pluralism in AI ethics and the need to avoid imposing homogenised “universal” values. Instead, ethical AI development should account for contextual diversity and local priorities to enhance both acceptance and fairness.

Practical implications in sensitive domains. In applied settings such as psychological counseling, education, and legal guidance, value misalignment may translate into recommendations that inadvertently conflict with users’ religious or cultural priorities (e.g., framing coping strategies, family obligations, or moral norms in ways that are culturally incongruent). This underscores the need for culturally informed guardrails, transparent disclosure of model limitations, and user education to prevent over-reliance on AI outputs in value-laden decisions.

### 4.8 Alignment with hypotheses

The results broadly supported the proposed hypotheses, revealing significant differences in values according to gender, academic level, and AI model type. Clear divergences were observed between humans and AI models, with religious values more salient among students and theoretical values dominating AI rankings. These findings reinforce the theoretical framework of Spranger’s classification and provide empirical evidence for its applicability in both human and AI contexts.

## 5 Conclusion

The present study contributes to the growing interdisciplinary dialogue on artificial intelligence, human values, and culturally situated meaning by examining value-related output patterns in large language models alongside the value priorities of university students. The findings point to a notable divergence between the dominant theoretical orientation observed in AI-generated responses and the stronger religious priority expressed by the human sample. This contrast is not merely descriptive; rather, it highlights the importance of cultural, linguistic, and educational context in the interpretation of value-related outputs generated by contemporary AI systems.

At a conceptual level, the study supports the cautious use of classical value frameworks, such as Spranger’s typology, in the analysis of AI-generated content. At the same time, the findings underscore the importance of interpretive discipline. What is being observed in large language models is better understood as patterned output under constrained prompting conditions, not as evidence of internal beliefs, intentions, or human-like value structures. This distinction is especially important in order to avoid anthropomorphic overreach and to preserve conceptual clarity in emerging psychological and interdisciplinary research on AI.

From an applied perspective, the results raise important considerations for educational, counseling, and other high-stakes contexts in which value-sensitive judgments matter. If AI systems are increasingly used to support reflection, communication, or decision-making in culturally grounded settings, then misalignment between model output patterns and human value priorities may have meaningful practical consequences. These findings therefore reinforce the need for culturally sensitive alignment practices, transparent prompt design, and more context-aware evaluation strategies.

At the same time, the study should be interpreted within its methodological boundaries, particularly the restricted human sampling frame and the model-specific nature of AI outputs. Further work should test the robustness and transferability of the observed patterns across broader samples, languages, and model families.

In view of these findings and their theoretical as well as practical implications, it is useful to move from interpretation to action-oriented guidance. The following recommendations therefore outline implications for users, researchers, and developers working with AI systems in culturally sensitive contexts.

### 5.1 Recommendations

For users of artificial intelligence systems, the findings of the present study highlight the importance of approaching AI-generated content with informed critical awareness, particularly in domains where cultural values, ethical judgments, and normative perspectives play a central role. While large language models can provide sophisticated and contextually coherent responses, their outputs may reflect patterns derived from training data and alignment procedures rather than culturally grounded human priorities. Users in educational, advisory, or decision-support contexts should therefore remain attentive to the interpretive limits of AI-generated responses and treat them as informational resources rather than authoritative value judgments.

For researchers, the results underscore the need for more systematic and cross-contextual investigations into value-related output patterns in artificial intelligence systems. Priority should be given to cross-cultural replication, multilingual validation, and robustness checks that examine whether the observed rankings remain stable under controlled variations in model and prompt conditions. Expanding methodological approaches that integrate psychological theory, cultural analysis, and computational evaluation will be especially important for advancing this emerging line of interdisciplinary inquiry.

For developers and designers of AI systems, the findings suggest the importance of integrating culturally sensitive alignment strategies and evaluation frameworks during model development and deployment. As AI technologies increasingly interact with users in socially meaningful and culturally diverse contexts, greater attention should be devoted to how training data, alignment processes, and prompting structures shape value-related output patterns. Designing AI systems that acknowledge cultural diversity, enhance transparency in response generation, and allow for context-aware adaptation may help mitigate potential mismatches between model outputs and human value expectations.

## Data Availability

The raw data supporting the conclusions of this article will be made available by the authors, without undue reservation.
