# Supplementary material for: Value systems of artificial intelligence and university students: theoretical dominance in large language models and religious priority in humans
Source: Front Psychol. 2026 Apr 21;17:1755145. doi: 10.3389/fpsyg.2026.1755145 (PMC13139343; doi:10.3389/fpsyg.2026.1755145)
Supplement: Supplementary file 1 [file Data_Sheet_1.docx]

Supplementary Tables

Supplementary Table S1. Rank-order stability across repeated LLM runs (within each model).

| Model | N (runs) | Kendall's W | χ² | df | p | Mean pairwise Spearman ρ | Worst-case Spearman ρ |
| --- | --- | --- | --- | --- | --- | --- | --- |
| ChatGPT-o1 | 7 | 0.802 | 28.083 | 5 | <0.001 | 0.769 | 0.462 |
| Gemini (code=2) | 5 | 0.448 | 11.192 | 5 | 0.048 | 0.311 | -0.058 |
| DeepSeek (code=3) | 5 | 0.84 | 21.012 | 5 | <0.001 | 0.8 | 0.585 |

Supplementary Table S2. Score dispersion across repeated LLM runs (within each model).

| Model | N (runs) | Dimension | Mean | SD | CV | 95% CI (low) | 95% CI (high) |
| --- | --- | --- | --- | --- | --- | --- | --- |
| ChatGPT-o1 | 7 | Religious | 26.0 | 5.89 | 0.23 | 20.55 | 31.45 |
| ChatGPT-o1 | 7 | Theoretical | 57.0 | 8.72 | 0.15 | 48.94 | 65.06 |
| ChatGPT-o1 | 7 | Economic | 29.57 | 8.32 | 0.28 | 21.87 | 37.27 |
| ChatGPT-o1 | 7 | Social | 46.86 | 6.31 | 0.13 | 41.02 | 52.69 |
| ChatGPT-o1 | 7 | Aesthetic | 33.86 | 6.15 | 0.18 | 28.17 | 39.54 |
| ChatGPT-o1 | 7 | Political | 35.14 | 6.09 | 0.17 | 29.51 | 40.78 |
| Gemini (code=2) | 5 | Religious | 23.4 | 10.36 | 0.44 | 10.54 | 36.26 |
| Gemini (code=2) | 5 | Theoretical | 40.2 | 8.04 | 0.2 | 30.21 | 50.19 |
| Gemini (code=2) | 5 | Economic | 25.2 | 9.31 | 0.37 | 13.64 | 36.76 |
| Gemini (code=2) | 5 | Social | 26.8 | 5.54 | 0.21 | 19.92 | 33.68 |
| Gemini (code=2) | 5 | Aesthetic | 27.2 | 9.52 | 0.35 | 15.37 | 39.03 |
| Gemini (code=2) | 5 | Political | 28.8 | 5.17 | 0.18 | 22.38 | 35.22 |
| DeepSeek (code=3) | 5 | Religious | 11.8 | 7.46 | 0.63 | 2.53 | 21.07 |
| DeepSeek (code=3) | 5 | Theoretical | 37.0 | 3.39 | 0.09 | 32.79 | 41.21 |
| DeepSeek (code=3) | 5 | Economic | 20.8 | 4.97 | 0.24 | 14.63 | 26.97 |
| DeepSeek (code=3) | 5 | Social | 26.0 | 3.74 | 0.14 | 21.35 | 30.65 |
| DeepSeek (code=3) | 5 | Aesthetic | 27.8 | 5.07 | 0.18 | 21.51 | 34.09 |
| DeepSeek (code=3) | 5 | Political | 27.4 | 2.88 | 0.11 | 23.82 | 30.98 |

**Appendix 1: AI Administration**

Appendix 1. AI Administration Protocol (Prompts and Settings)

This appendix documents the standardised protocol used to administer the Arabic version of the Study of Values (SOV) to the large language models (LLMs).

A1. Models and access

- ChatGPT-o1 (OpenAI): Accessed via the official ChatGPT web interface.
- Gemini-2.0 (Google): Accessed via the official Gemini web interface.
- DeepSeek-V3 (DeepSeek): Accessed via the official DeepSeek interface.

All models were accessed from the same physical location and network environment during the period 1 December 2024 to 30 January 2025.

A2. Language and session structure

- All prompts and SOV items were presented in Arabic, using the same wording as in the human version.
- For each run, a new conversation was started with the model; no prior context was included.
- At the start of each run, the model received a task instruction prompt (see A3).
- The 45 SOV items were then presented one at a time, each with two answer options labelled (A) and (B).
- The model was instructed to answer with a single letter (A or B) for each item.

A3. Task instruction prompt (translated from Arabic)

“You are a large language model. In this task, you will be presented with a series of items from a psychological values questionnaire. For each item, you will see two options labelled (A) and (B). Your task is to choose the option most consistent with the response pattern generated under these task instructions. For every item, respond with only one letter: A or B. Do not provide explanations unless explicitly asked. Do you understand?”

After the model confirmed understanding, the SOV items were administered sequentially.

A4. Item presentation format (example)

*Item X (example structure translated from Arabic):*

“Please choose the option that is most consistent with the response pattern generated under these task instructions:

(A) [Arabic text of option A]
(B) [Arabic text of option B]

Respond with only one letter: A or B.”

This structure was used for all 45 SOV items.

A5. Model settings and response control

- When adjustable, the temperature parameter was set to a low value (≈ 0.2) to reduce randomness and enhance response stability.
- Default values were used for other parameters (e.g., top-p, maximum tokens), as these did not materially affect the short A/B responses.
- If a model produced an answer other than a single letter (e.g., “I think A is better because…”) the following control prompt was sent:

“Please answer with only one letter (A or B), without any explanation.”

- If the model still failed to comply after two prompts, the response was coded as missing for that item and excluded from scoring.

A6. Number of runs and scoring

- ChatGPT-o1: 7 complete runs (all 45 items answered in the correct format).
- Gemini-2.0: 5 complete runs.
- DeepSeek-V3: 5 complete runs (additional attempts were discarded when the model did not comply with the response format).

For each run, A/B responses were converted to SOV domain scores using the standard scoring key. This yielded six domain scores (theoretical, religious, social, political, economic, aesthetic) per run and per model. Across runs, these scores were used to compute descriptive statistics and to estimate the stability of each model’s value pattern (see Section 2.4).

### **Appendix 2: Correlation Values Between the Dimensions and Their Corresponding Value Domains**

## **Economic value:**

| paragraph | Correlation coefficient | Significance Statistical significance |
| --- | --- | --- |
| 1 | 82.39 | *** |
| 2 | 83.88 | *** |
| 3 | 82.06 | *** |
| 4 | 84.53 | *** |
| 5 | 83.35 | *** |
| 6 | 86.03 | *** |
| 7 | 82.32 | *** |
| 8 | 81.25 | ** |
| 9 | 82.59 | *** |
| 10 | 82.78 | *** |
| 11 | 84.48 | *** |
| 12 | 81.84 | *** |
| 13 | 81.39 | ** |
| 14 | 85.21 | *** |
| 15 | 82.78 | *** |
| 16 | 83.37 | *** |
| 17 | 81.39 | ** |
| 18 | 81.25 | ** |
| 19 | 82.59 | *** |
| 20 | 83.88 | *** |

**Social value**

| No. Poverty | Correlation coefficient | Significance Statistical significance |
| --- | --- | --- |
| 1 | 80.22 | * |
| 2 | 80.58 | * |
| 3 | 83.60 | *** |
| 4 | 91.52 | *** |
| 5 | 95.53 | *** |
| 6 | 81.43 | ** |
| 7 | 82.32 | *** |
| 8 | 82.28 | *** |
| 9 | 82.19 | *** |
| 10 | 82.19 | *** |
| 11 | 82.65 | *** |
| 12 | 82.08 | *** |
| 13 | 82.78 | *** |
| 14 | 93.21 | *** |
| 15 | 83.77 | *** |
| 16 | 82.07 | *** |
| 17 | 82.96 | *** |
| 18 | 82.25 | *** |
| 19 | 82.18 | *** |
| 20 | 81.11 | ** |

**Aesthetic value**

| No. Poverty | Correlation coefficient | Significance Statistical significance |
| --- | --- | --- |
| 1 | 80.22 | * |
| 2 | 84.22 | *** |
| 3 | 83.95 | *** |
| 4 | 84.89 | *** |
| 5 | 83.37 | *** |
| 6 | 86.03 | *** |
| 7 | 85.09 | *** |
| 8 | 82.51 | *** |
| 9 | 86.28 | *** |
| 10 | 84.50 | *** |
| 11 | 86.60 | *** |
| 12 | 86.24 | *** |
| 13 | 83.81 | *** |
| 14 | 81.57 | ** |
| 15 | 97.11 | *** |
| 16 | 83.01 | *** |
| 17 | 77.47 | – |
| 18 | 91.16 | *** |
| 19 | 80.36 | * |
| 20 | 92.57 | *** |

**Religious value**

| No. Poverty | Correlation coefficient | Significance Statistical significance |
| --- | --- | --- |
| 1 | 81.79 | *** |
| 2 | 86.40 | *** |
| 3 | 81.59 | ** |
| 4 | 83.58 | *** |
| 5 | 84.45 | *** |
| 6 | 84.91 | *** |
| 7 | 84.55 | *** |
| 8 | 93.68 | *** |
| 9 | 86.08 | *** |
| 10 | 85.76 | *** |
| 11 | 81.86 | *** |
| 12 | 88.00 | *** |
| 13 | 84.40 | *** |
| 14 | 85.44 | *** |
| 15 | 86.17 | *** |
| 16 | 80.15 | * |
| 17 | 82.42 | *** |
| 18 | 85.34 | *** |
| 19 | 82.92 | *** |
| 20 | 85.41 | *** |

**Political value**

| No. Poverty | Correlation coefficient | Significance Statistical significance |
| --- | --- | --- |
| 1 | 83.95 | *** |
| 2 | 78.16 | – |
| 3 | 82.35 | *** |
| 4 | 85.00 | *** |
| 5 | 81.57 | ** |
| 6 | 85.46 | *** |
| 7 | 82.69 | *** |
| 8 | 83.97 | *** |
| 9 | 82.63 | *** |
| 10 | 80.45 | * |
| 11 | 82.42 | *** |
| 12 | 81.59 | ** |
| 13 | 81.13 | ** |
| 14 | 80.08 | * |
| 15 | 83.93 | *** |
| 16 | 84.00 | *** |
| 17 | 81.20 | ** |
| 18 | 81.57 | ** |
| 19 | 85.19 | *** |
| 20 | 82.42 | *** |

**Theoretical value**

| No. Poverty | Correlation coefficient | Significance Statistical significance |
| --- | --- | --- |
| 1 | 78.46 | – |
| 2 | 78.61 | – |
| 3 | 69.00 | *** |
| 4 | 76.99 | – |
| 5 | 79.01 | – |
| 6 | 78.29 | – |
| 7 | 77.68 | – |
| 8 | 72.45 | *** |
| 9 | 75.77 | – |
| 10 | 69.32 | *** |
| 11 | 73.76 | ** |
| 12 | 78.29 | – |
| 13 | 72.84 | *** |
| 14 | 76.78 | – |
| 15 | 71.77 | *** |
| 16 | 76.76 | – |
| 17 | 74.31 | ** |
| 18 | 76.67 | – |
| 19 | 81.61 | ** |
| 20 | 74.61 | * |

### **Appendix 3: Post Hoc Tests for the Theoretical Value According to Academic Level**

Multiple Comparisons (Tukey HSD)

| **Multiple Comparisons** | | | | | | |
| --- | --- | --- | --- | --- | --- | --- |
| Dependent Variable: NALlend2 | | | | | | |
| Tukey HSD | | | | | | |
| (I) Stage | (J) Stage | Mean Difference (I-J) | Std. Error | Sig. | 95% Confidence Interval | |
|  |  |  |  |  | Lower Bound | Upper Bound |
| Bachelor | Master | -1.7384- | 1.22942 | .336 | -4.6405- | 1.1638 |
|  | Ph.D. | -4.3304-^*^ | 1.17067 | .001 | -7.0939- | -1.5669- |
| Bachelor | Master | 1.7384 | 1.22942 | .336 | -1.1638- | 4.6405 |
|  | Ph.D. | -2.5920- | 1.17551 | .073 | -5.3670- | .1829 |
| Bachelor | Master | 4.3304^*^ | 1.17067 | .001 | 1.5669 | 7.0939 |
|  | Ph.D. | 2.5920 | 1.17551 | .073 | -.1829- | 5.3670 |

```

Homogeneous Subsets (Tukey HSD) **Homogeneous Subsets**

## Table showing the descriptive statistics of students' theoretical value according to gender and stage

| **Theoretical value** | | | |
| --- | --- | --- | --- |
| Tukey HSD^a,b,c^ | | | |
| Stage | N | Subset | |
|  |  | 1 | 2 |
| Bachelor | 67 | 40.7313 |  |
| Master | 66 | 42.4697 | 42.4697 |
| Ph.D. | 81 |  | 45.0617 |
| Sig. |  | .313 | .078 |

| Means for groups in homogeneous subsets are displayed.  Based on observed means.  The error term is Mean Square(Error) = 50.253. |
| --- |
| a. Uses Harmonic Mean Sample Size = 70.717. |
| b. The group sizes are unequal. The harmonic mean of the group sizes is used. Type I error levels are not guaranteed. |
| c. Alpha = .05. |

### **Appendix 4: Kruskal–Wallis Test for Comparing the AI Models**

Test Statistics (Kruskal-Wallis)

| **Test Statistics^a,b^** | | | | | | |
| --- | --- | --- | --- | --- | --- | --- |
|  | Theoretical value | Social value | Economic value | Political value | Aesthetic value | Religious value |
| Kruskal-Wallis H | 10.145 | 10.053 | 2.006 | 5.089 | 5.990 | 1.416 |
| df | 2 | 2 | 2 | 2 | 2 | 2 |
| Asymp. Sig. | .006 | .007 | .367 | .079 | .050 | .493 |

### **Combined Descriptive Statistics for the AI Models**

| **Descriptive Statistics** | | | | | |
| --- | --- | --- | --- | --- | --- |
|  | N | Mean | Std. Deviation | Minimum | Maximum |
| Theoretical value | 14 | 45.64 | 13.454 | 27 | 72 |
| Social value | 14 | 36.43 | 11.837 | 22 | 55 |
| Economic value | 14 | 27.00 | 7.845 | 15 | 40 |
| Political value | 14 | 30.57 | 7.633 | 16 | 48 |
| Aesthetic value | 14 | 30.00 | 7.463 | 19 | 45 |
| Religious value | 14 | 22.21 | 8.997 | 5 | 35 |

### **Appendix 5: One-Sample t-Test Results Examining the Differences Between Students’ Means and the Means of AI Models, Presented Separately for Each Model**

##

| Sig. (2-tailed) | df | t | Std. Deviation | Mean  Students | The hypothetical "average intelligence" |  | Value |
| --- | --- | --- | --- | --- | --- | --- | --- |
| .000 | 213 |  | 7.45 | 43 | 46.176 | General | Theoretical |
| < .001 |  | -27.67 | 7.45 | 42.90 | 57.0000 | GPT |  |
| .000 |  | 5.315 | 7.45 | 42.90 | 40.2000 | Gemini |  |
| 000 |  | 11.60 |  |  | 37.0000 | DeepSeek |  |
| < .001 | 213 | 5.248 | 6.184 | 37.748 | 35.529 | General | social |
| .000 |  | -21.886- |  |  | 47.0000 | GPT |  |
| .000 |  | 20.692 |  |  | 29.0000 | Gemini |  |
| .000 |  | 27.789 |  |  | 26.0000 | DeepSeek |  |
| < .001 | 213 | 31.115 | 7.125 | 40.860 | 31.000 | General | Economical |
| .000 |  | 23.181 |  |  | 29.5714 | GPT |  |
| .000 |  | 32.154 |  |  | 25.2000 | Gemini |  |
| .000 |  | 41.188 |  |  | 20.8000 | DeepSeek |  |
| < .001 | 213 | 23.245 | 6.599 | 41.486 | 30.118 | General | political |
| .000 |  | 14.068 |  |  | 35.1429 | GPT |  |
| .000 |  | 28.122 |  |  | 28.8000 | Gemini |  |
| .000 |  | 31.226 |  |  | 27.4000 | DeepSeek |  |
| < .001 | 213 | 6.078 | 7.881 | 33.393 | 25.706 | General | Aesthetic |
| .000 |  | -.923- |  |  | 33.8571 | GPT |  |
| .000 |  | 11.494 |  |  | 27.2000 | Gemini |  |
| .000 |  | 10.380 |  |  | 27.8000 | DeepSeek |  |
| < .001 | 213 | 37.234 | 8.859 | 43.607 | 21.059 | General | Religious |
| .000 |  | 29.075 |  |  | 26.0000 | GPT |  |
| .000 |  | 33.368 |  |  | 23.4000 | Gemini |  |
| .000 |  | 52.523 |  |  | 11.8000 | DeepSeek |  |

إ

### **Appendix 6: Ranking of the Six Values Based on Mean Scores for Both AI Models and University Students**

| Values | IQ models | | | Gender | | School stage | | |
| --- | --- | --- | --- | --- | --- | --- | --- | --- |
|  | GPT | Gemini | DeepSeek | Male | Female | BuckAloysius | Master | Ph.D. |
| Theoretical | 57.000 | 40.2000 | 37.00 | 43.45 | 41.18 | 40.73 | 42.47 | 45.06 |
| Social | 47.000 | 29.0000 | 26.00 | 37.127 | 37.127 | 36.627 | 38.318 | 38.210 |
| economic | 29.571 | 25.2000 | 20.80 | 41.276 | 40.163 | 40.358 | 40.667 | 41.432 |
| Political | 35.142 | 28.8000 | 27.40 | 41.955 | 40.700 | 43.433 | 40.848 | 40.39 |
| Aesthetic | 33.857 | 27.2000 | 27.80 | 33.052 | 33.962 | 35.746 | 33.318 | 31.506 |
| Religious | 26.000 | 23.4000 | 11.80 | 42.649 | 45.212 | 43.104 | 44.379 | 43.395 |
